# Parent experiences of infant feeding in congenital hyperinsulinism: a thematic analysis

**DOI:** 10.3389/fendo.2026.1955679

**Published:** 2026-10-20

**Authors:** Sarah Worthington, Eleanor Ginbey, Osarugue Otabor, Caroline Hall, Bethany Allchin, Sameera Hannah Auckburally, Maria Salomon-Estebanez, Indraneel Banerjee, Chris Worth

**Affiliations:** 1 Department of Paediatric Endocrinology, Royal Manchester Children’s Hospital, Manchester, United Kingdom; 2 Department of Paediatric Dietetics, Royal Manchester Children’s Hospital, Manchester, United Kingdom; 3 Department of Health Professions, Faculty of Health and Education, Manchester Metropolitan University, Manchester, United Kingdom; 4 Faculty of Biology, Medicine and Health, University of Manchester, Manchester, United Kingdom

**Keywords:** CHI, congenital hyperinsulinism, feeding, feeding aversion, hypoglycaemia

## Abstract

**Introduction:**

Congenital Hyperinsulinism (CHI) is a rare condition of hypoglycaemia due to excess insulin secretion. Feeding problems are frequent, and often persistent, in patients with CHI. The range, extent and persistence of feeding problems has been described, but parent and family perspectives are lacking. As feeding is a key aspect of the maintenance of glycaemic stability, and a vital part of infant development and family bonding, it is important to understand the impact of feeding problems on parents and caregivers. This qualitative study explored caregivers’ experiences of feeding during the initial years following diagnosis.

**Methods:**

We conducted semi-structured interviews with caregivers of patients with CHI, from a single international centre of excellence. Patients had received a confirmed genetic diagnosis of CHI between 2015 and 2025. All interviews were completed using Microsoft Teams, recorded and transcribed. Transcriptions were analysed using thematic analysis to identify themes.

**Results:**

Thematic analysis identified four main themes; Theme 1: Medical Management Disrupts Feeding: Frequent feeding schedules, enteral tube feeding and medication regimens were perceived to interfere with the establishment of typical feeding behaviours and family mealtime routines. Theme 2: Feeding Aversion and Delayed Oral Development: Parents described feeding aversion and delays in oral feeding skill development, with many children demonstrating reduced interest in eating, reluctance to try new foods and prolonged dependence on enteral feeding. Theme 3: Feeding Driven by Glycaemic Control: Feeding was consistently described as being targeted to maintain glycaemic stability rather than respond to hunger and satiety cues. Theme 4: Psychosocial Impact on Families: Feeding challenges had a substantial psychosocial impact on families, contributing to parental stress and anxiety, increasing caregiver burden, and affecting family routines, social activities and confidence around mealtimes.

**Conclusion:**

This study identified the complex, demanding and distressing challenges faced by families of infants with CHI, adversely affecting family wellbeing. Early multidisciplinary input, including dietetics, speech and language therapy, and psychological support, along with practical CHI-specific feeding guidance may reduce burden and improve outcomes. The findings of this study highlight the need for training and education in local healthcare teams managing CHI.

## Introduction

Congenital Hyperinsulinism (CHI) is a rare condition which is characterised by the unregulated release of insulin by the pancreatic β-cells, causing severe and profound hypoglycaemia (1, 2). Hypoglycaemia refers to the blood glucose levels being below the normal range of 4mmols and 6mmols (3). In CHI, there is a disruption of glucose-dependent feedback mechanisms managing insulin secretion which results in persistent, unregulated insulin release and episodes of severe, unpredictable hypoglycaemia (4).

CHI can be classified as transient or genetic. Transient CHI is associated with risk factors such as perinatal stress, intrauterine growth restriction and maternal diabetes and does not have an identified genetic component (5). Transient forms of CHI often resolve in days to weeks (6). Genetic CHI can be caused by a single mutation in the insulin secretory pathway or via genes which cause syndromes (such as Beckwith Wiedemann Syndrome) (7) and tends to be more persistent. The incidence of genetic CHI is approximately 1:28,000 births, in the UK (8), with higher rates in consanguineous populations. Infants with both transient and genetic type CHI are at risk of hypoglycaemia related brain injuries (6).

Infants with CHI, both transient and genetic, often require extensive periods of admission in hospital. During admission, there are multiple risk factors for the development of feeding problems (9). Patients often require intensive pharmacological therapies and supraphysiological carbohydrate provision to maintain glycaemia (10). Many infants with CHI often require high concentration intravenous glucose, or parenteral nutrition (PN). This often results in extended periods of non-oral feeding which contribute to difficulties with feeding (11). As such, in order to provide nutrition, many patients require the use of a nasogastric tube (NGT) or a percutaneous endoscopic gastrostomy (PEG). A nasogastric tube is a temporary feeding tube used to provide enteral nutrition; this can later be exchanged for a PEG following discussions with families and clinicians, if feeding remains challenging. Unfortunately, tube feeding often reduces oral feeding and increases the risk of feeding problems (9). Children with CHI often require treatment with diazoxide. Diazoxide is known to have an unpleasant taste and can have gastrointestinal side effects such as vomiting and nausea (12). In those unresponsive to diazoxide second-line treatment is subcutaneous Octreotide, which also comes with gastrointestinal side effects such as gastrointestinal dysmotility (13). Both medications thus increase the possibility of feed aversion and other feeding problems.

Due to the cumulative effect of the issues described above, feeding issues/oral aversions are considered a frequent problem in patients with CHI (10, 14). Feeding difficulties have been noted to effect approximately 31-45% of CHI patients, with patients having issues with vomiting, impaired sucking and swallowing, and feed aversion (10). It is recognised that feeding difficulties are a major cause of anxiety and stress among parents of infants/children with CHI (15).

While previous studies have addressed the causes and rates of feeding difficulties in children with CHI, there has been little attention given to the experiences of families. Given the high incidence of feeding difficulties in CHI, and the limited evidence base, this study aimed to explore the experiences of parents/caregivers of children with CHI, specifically in relation to feeding challenges. This aimed to identify key themes in order to inform clinical support and future service development for families.

## Aims

1. To explore the experiences of parents of children with congenital hyperinsulinism (CHI), with a focus on feeding and related challenges.
2. To examine the impact of any feeding challenges on daily life, caregiving burden, and family functioning.

## Methods

Patients were recruited from families receiving care through a single international centre of excellence. Eligible participants were parents or primary caregivers of children with a confirmed genetic form of CHI diagnosed within 10 years. A pre-existing database of genetic results held by the service was used to identify eligible families.

A random sampling approach was employed to reduce selection bias and ensure that participants were not chosen on the basis of disease severity, treatment pathway, or clinician familiarity. Each eligible family was allocated a unique study number, and numbers were selected at random to identify families to be approached for participation.

Of the 38 families who met the eligibility criteria, 18 families were invited to participate. Initial contact was made by telephone by either the principal investigator, a CHI Specialist Nurse, or a doctor within the CHI service. Families who expressed interest were sent the participant information sheet and consent form by email. Interviews were scheduled according to parental preference, and participants were given a minimum of one week between receipt of the study information and the interview date to consider participation.

Recruitment was ceased once the target sample size of 10 interviews was reached, this sample size was primarily determined based on the concept of information power. This sample size was based on the high specificity and homogeneity of the sample cohort, which increases the information power of each individual interview and the anticipated depth and richness of interview data, which was also supported by the interviewers pre-existing clinical relationship with participating families. This sample size also reflected practical feasibility of undertaking in-depth qualitative interviews within the timeframe and resources available for this study. As this study was exploratory, sample size was not determined using statistical power calculations or the concept of data saturation. Instead, recruitment aimed to obtain information-rich accounts from parents of children with congenital hyperinsulinism. Given the specificity of the research question, the rarity of the condition, and the depth of the semi-structured interviews, the sample was considered sufficient to generate meaningful patterns of shared experience.

Ethical approval for the study protocol, consent documentation, and interview topic guide was obtained from the Health Research Authority (REC reference: HRA 07/H1010/88). Parents/care givers provided written consent when consenting to participate in the interview process.

Following informed consent, interviews were conducted and recorded via Microsoft Teams^®^. Written informed consent was obtained from all participants prior to participation. At the commencement of each interview, verbal confirmation of consent was obtained and recorded. Participants were informed of the purpose of the study, the voluntary nature of participation, and their right to withdraw at any stage without consequence.

Data were collected through semi-structured interviews designed to explore parental experiences of infant feeding in children with CHI. Interviews were conducted remotely using Microsoft Teams by two members of the research team: a designated CHI nurse specialist or a doctor, and a non-specialist paediatric dietitian. The semi-structured format was guided by a predefined topic guide covering three principal domains: feeding experiences, factors affecting feeding, and the impact on the child and family. Participants were invited to expand on their responses, and iterative probing was employed to explore relevant issues in greater depth as they arose. In cases where both parents wished to take part, joint interviews were conducted. Each interview lasted 40–60 minutes. Interview recordings were transcribed using the transcription function within Microsoft Teams. To ensure accuracy, each recording was reviewed alongside the generated transcript, and corrections were made where necessary. Transcripts were subsequently proofread to ensure they accurately reflected the content and context of the discussions.

Familiarisation of the data was achieved through repeated reading and noting of initial impressions within the transcripts. Due to the lack of any literature regarding family experiences of feeding in CHI, a predominantly inductive coding approach was used, with themes derived directly from the data rather than a pre-established framework.

NVivo software was used to apply initial codes to the dataset. These were then combined to develop themes representing recurring patterns in participants’ experiences. Themes were refined through review of all relevant data extracts before being finalised and named to ensure sufficient support from the data.

Interview transcripts were analysed using Braun and Clarke’s reflexive thematic analysis, using a predominantly inductive approach (16). Coding and analysis were completed by a CHI Specialist Nurse who also conducted the interviews. In line with a reflexive thematic analytical approach, coding and theme development was undertaken by a single analyst; with existing clinical relationships with participating families, but no prior background in feeding research specifically. In line with the principles of reflexive thematic analysis, the researcher’s clinical knowledge of CHI and established rapport with families was considered integral to the analytical process rather than a source of bias. Codes and theme development was guided by patterns of meaning within participant accounts and discussed with co-authors to support interpretive depth and to challenge initial interpretations, but final analytical decisions rested with the primary analyst whose aim, at all times, was to allow for the emergence of the authentic family voice as much as possible.

The position of the CHI Specialist Nurse supported the analytical process, the clinical familiarity likely sensitized the analyst to clinically meaningful aspects of the accounts provided by participants, this prior relationship may have shaped rapport and disclosure during the interview process. Reflexive engagement with this positionality was maintained throughout the analysis using analytic memos. These relational dynamics were considered as part of the reflexive interpretation of the data and monitored throughout data collection and analysis, and reflexive memos were utilised to reflect how clinical knowledge and relational closeness to the participants shaped the interpretive decisions.

## Results

Eighteen families were approached to participate in the study: four declined participation; four were not contactable by phone; and ten consented and participated. Ages of the participants ranged from 6 months to 9 years of age with a median age of 34 months. Details of interviewees, and demographic data, are provided in **Table 1**. Of the 18 families invited, 4 declined participation. A further 4 families could not be contacted despite telephone attempts. The final sample comprised 10 participants who completed an interview.

**Table 1:** Patient demographics with gender, genetic mutation and treatment course.

Patient	Interview participant	Age at the time of interview	Gender	Genetics	Treatment	Length of stay	Method of feeding:
1	Mother	9 months	Male	Homozygous ABCC8	Subtotal Pancreatectomy & Octreotide	107	PEG, continuous & oral tastes
2	Mother	11 months	Male	Beckwith Wiedmann Syndrome	Subtotal Pancreatectomy & Diazoxide	211	PEG, continuous & oral tastes
3	Mother	4 years	Female	Paternally inherited Heterozygous KCNJ11	Focal Lesionectomy	92	Nasogastric tube & oral tastes
4	Mother	14 months	Male	Homozygous ABCC8	Subtotal Pancreatectomy & Octreotide	107	PEG, continuous & oral tastes
5	Mother	6 months	Male	HNF4A	Diazoxide	69	Nasogastric tube & Oral feeding
6	Mother	20 months	Female	HNF1A	Diazoxide	65	Nasogastric tube & Oral feeding
7	Mother	4 years	Female	Paternal Heterozygous ABCC8	Focal Lesionectomy	87	Nasogastric tube & Oral feeding
8	Both	5 years	Male	Compound Heterozygous ABCC8	Dasiglucagon	61	PEG, continuous & oral tastes
9	Both	9 years	Female	Paternally Heterozygous KCNJ11	No treatment – Previously Diazoxide	107	Nasogastric tube & Oral feeding
10	Mother	6 years	Male	Heterozygous ABCC8	Focal Lesionectomy	58	Nasogastric tube & Oral feeding

The study cohort comprised patients with genetically confirmed CHI, representing a spectrum of disease severity. Management strategies included medical treatment with diazoxide, surgical resection of a focal pancreatic lesion (focal lesionectomy), and subtotal pancreatectomy for patients with diffuse disease. Patients who underwent subtotal pancreatectomy required ongoing treatment with octreotide postoperatively because of persistent hypoglycaemia.

The duration of inpatient admission ranged from 58 to 211 days, reflecting the complexity of disease management. The inpatient admission length for patients who underwent pancreatic surgery ranged from 58 days to 211 days, with the length of stay for patients who did not require surgery being 65 days to 69 days. Prolonged hospitalisation has been recognised as a contributing factor to the development of feeding difficulties and feeding aversion in infants with CHI and therefore represents an important characteristic of the study population.

Four major themes emerged from the data, after thematic analysis. These are summarised sequentially.

1. Medical Management Disrupts Feeding.
2. Feeding Aversion and Delayed Oral Development.
3. Feeding Driven by Glycaemic Control.
4. Psychosocial Impact on Families.

Each theme is introduced and summarised and selected quotations are used to provide evidence for this theme, highlight specific points and ensure accurate representation of the patient voice. **Table 2** provides an overview of the main themes, describing families’ experiences of feeding in infants with CHI.

**Table 2:** Description of four major themes and subthemes in families’ experiences of feeding in CHI.

Theme	Medical management disrupts feeding	Feeding aversion and delayed oral development	Feeding driven by glycaemic control	Psychosocial impact on families
Theme description	This theme reflects how parents view and manage feeding as a critical part of their child’s medical care, requiring careful planning to maintain glucose stability.	This theme captures how CHI-related feeding difficulties interfere with the child’s normal feeding development, often leading to food refusal or aversion.	This theme highlights the emotional and practical strain on parents who must maintain constant vigilance to manage their child’s feeding and prevent hypoglycaemic episodes.	This theme reflects how feeding challenges affect family routines, daily activities, and social interactions, often limiting normal participation for both child and family.
Subtheme 1	Early disruption to feeding establishment	Delayed feeding milestones	Feeding to maintain blood glucose	Emotional burden and parental distress
Subtheme 2	Negative oral experiences from medical interventions	Oral aversion and food refusal	Fear of hypoglycaemia influencing feeding	Restrictions on family and social life
Subtheme 3	Treatment-related effects on feeding	Dependence on enteral feeding	Balancing oral intake with nutritional needs	Constant vigilance and caregiver burden
Subtheme 4	Feeding driven by medical requirements	Inconsistent feeding progression	Feeding becoming a clinical task	Need for specialist support and education

### Medical management disrupts feeding

Parents consistently described how the medical management of CHI fundamentally altered the establishment of early feeding. Extended hospital admissions, periods of fasting, enteral feeding, frequent blood glucose monitoring and strict feeding schedules disrupted opportunities for infants to develop typical feeding behaviours. Parents reflected that these necessary medical interventions often shaped their child’s relationship with feeding from birth, creating challenges that persisted beyond the neonatal period.

Several parents described prolonged hospitalisation as interrupting normal feeding development. Extended admissions often coincided with periods when infants were nil by mouth or received minimal or limited oral feeds, with emphasis on intravenous fluid and nutrition, reducing opportunities to establish breastfeeding or bottle feeding and delaying progression towards age-appropriate feeding.

‘I think most of it is because we spent nearly 4 months in hospital. He spent a few weeks nil by mouth. And from birth for a few weeks, he was only allowed like 10-15mls every two to three hours.’ - P1.

Some parents believed that continuous glucose management and the medical need to avoid and reduce hypoglycaemia limited their child’s opportunity to experience hunger. They perceived this as disrupting the development of intrinsic feeding cues and motivation to feed, with consequences extending into later feeding behaviours.

‘He didn’t need to work to get his drink. He didn’t know hunger because he was never allowed to be hungry because his blood sugars were being monitored and maintained all the time. So, he just didn’t have that drive or that absolute innate hunger that a baby would have to just feed.’ - P10.

Parents often interpreted subsequent feeding difficulties as a consequence of the medical management required during the neonatal period and early infancy. They described prolonged hospitalisation and repeated interventions as “blockers” that interrupted feeding progression and contributed to delayed feeding development.

‘I think he has had a lot of blockers that have stopped him from progressing. Like he was in hospital for so long.’ - P2.

Beyond the impact on infants in the hospital, parents described the complexity of delivering treatment at home. In keeping with the hospital focus on hypoglycaemia prevention, parents often had to accept a variety of mechanical feeding strategies such as nasogastric or gastrostomy tube feeding. Feeding became a technically demanding process that extended beyond preparing and offering milk. Families described learning to manage nasogastric tubes, checking tube placement, modifying feeds and administering nutritional supplements, transforming feeding into a highly medicalised task that differed substantially from their expectations of caring for a newborn.

‘Our original plan we knew we were going to give formula and bottles, but we thought she was going to feed like a normal baby. And then everything happened, and we had to learn how to use the NG tube. Then we had to try different formulas because she wasn’t tolerating the milk, and we had to use the Polycal in feeds too. It was a lot harder than we expected, because we had to do all these extra steps. We had to use the NG tube, check the aspirate and the PH level. So, it was just a lot harder. It was a lot to learn.’ - P6.

Collectively, these accounts demonstrate how medical management disrupted the expected experience of infant feeding. Parents anticipated a natural, nurturing and developmental aspect of early parenthood, which became dominated by clinical demands and treatment related priorities. Due to this, the context of feeding was experienced less as a normal interaction between parent and baby to a medically directed task, which disrupted both the development of feeding and parental experience of caring for their baby through feeding.

### Feeding aversion and delayed oral development

Parents described feeding difficulties as extending beyond the neonatal period, with many reporting persistent oral aversion, delayed progression through expected feeding milestones and prolonged reliance on enteral feeding. Rather than feeding improving as infants matured, parents often experienced feeding development as unpredictable, characterised by limited oral intake, vomiting and refusal to feed. These challenges were frequently interpreted as consequences of the medical management required to maintain glycaemic stability during infancy.

Many parents described oral feeding progressing more slowly than they had anticipated. Infants were often unable to complete bottle feeds because of gagging or vomiting, requiring the remainder of feeds to be administered via a nasogastric tube or gastrostomy to prevent hypoglycaemia. Parents contrasted these experiences with feeding development in their other children, highlighting how CHI altered their expectations of normal infant feeding.

‘He’s making progress but it’s not what I expected, because what I’m used to, my children before were taking full bottles. But with P1 he gets to maybe half of his feed and he starts to show signs that he is going to vomit, sometimes he does vomit. Sometimes he is gagging, which is not what I expected. Then I have to give the rest of his feed by the gastrostomy. Because otherwise his sugar will go down.’- P1.

Parents also described oral intake deteriorating over time rather than improving. Several reported that infants gradually accepted smaller feed volumes before refusing bottles altogether, delaying the transition towards complementary feeding and creating uncertainty around feeding progression.

‘At around 5 months old it started getting worse, to the point he was only having 5 or 10mls and then he just stopped taking the bottle. We pretty much stopped bottles until we started trying to do weaning with him. Which sometimes he will have and sometimes he won’t.’ - P2.

A recurring feature of parents’ reflections was the tension between encouraging oral feeding and maintaining blood glucose levels. Parents described feeling unable to offer opportunities for developmental feeding because prescribed feeding schedules left infants too full to explore complementary foods. At the same time, they feared that offering additional feeds could trigger vomiting, reducing nutritional intake and compromising glycaemic stability. As a result, parents experienced feeding as a constant negotiation between supporting oral development and prioritising metabolic control.

‘Because the focus is on three hourly feeds, there’s not a chance for me to wean him and give him food because he’s still full. And then there’s the fear of if I try to give him feeds in between, I’m scared he’s going to vomit his feeds. And he needs his feed to keep his sugars stable. It’s trying to find a balance, but then unable to find a balance, because there’s no way.’ - P4.

For some parents, repeated episodes of vomiting and enteral feeding changed the emotional experience of feeding. Rather than viewing feeding as a positive interaction, parents described anticipating distress with every feed and feeling that nutritional requirements took precedence over their infant’s willingness or readiness to feed. This sometimes resulted in feeding being perceived as forceful or coercive.

‘She would drink around 10mls of the amount she was due, then the rest was given by the NG tube. As soon as it went down the NG tube, within minutes she would be sick and bring it all back up. It felt like we were force feeding her. That’s what it felt like. I would actually dread feeding her because I knew what was going to happen every time we fed her.’ - P3.

Collectively, these accounts demonstrate that feeding aversion was experienced not as an isolated feeding problem but as part of a broader disruption to oral feeding development. Parents described how the need to prioritise glycaemic stability often conflicted with opportunities to develop typical feeding skills, resulting in prolonged dependence on enteral feeding, delayed feeding milestones and emotionally challenging feeding interactions.

### Feeding driven by glycaemic control

Parents consistently described feeding decisions as being governed by the need to maintain safe blood glucose levels rather than responding to their infant’s appetite or developmental readiness. At hospital and later at home, parents’ actions were influenced by the underlying medical need to prevent hypoglycaemia. Therefore, parents’ expectations migrated from perceiving feeding as a natural activity to accepting feeding as a medical intervention driven by the need to maintain normal glycaemic status, with nutritional intake prioritised over hunger cues, food preferences or typical feeding approaches. As a result, parents described adapting their feeding practices in ways they felt differed fundamentally from conventional infant feeding. The underlying principle of hypoglycaemia prevention guided parents to learn and accept feeding driven by the need to achieve glycaemic control. This was more frequently described in the home environment.

‘When we were in hospital, he couldn’t even last two hours, 45 minutes without a feed. As he was getting older, maybe normally if a baby didn’t have Hyperinsulinism they wouldn’t be feeding that often. It was just kind of as soon as he started getting low, we had to just feed him.’ - P5.

Several parents contrasted their experiences with those of feeding previous children. While refusal of feeds had previously been viewed as a normal part of infant behaviour, the presence of CHI removed this flexibility. Parents described feeling that every prescribed feed had to be completed regardless of whether their infant appeared hungry or willing to feed, creating uncertainty about the longer-term impact this might have on their child’s relationship with food.

‘Previously with our other two, if they weren’t drinking their bottles that was fine, they just wouldn’t have a bottle that day. But when you have one with CHI that’s just not an option, they just have to have it. So, I don’t know what effect that might have, when you’re not hungry and you don’t want it. But he is still being forced to have it. What kind of effect that might have.’ - P2.

Parents frequently described feeling compelled to encourage or complete feeds despite resistance from their infant because maintaining adequate nutritional intake was essential for glycaemic stability. This altered the emotional experience of feeding, with parents describing discomfort, anxiety and anticipation of hypoglycaemia whenever feeds were unsuccessful. Feeding therefore became associated with fear rather than a positive parent–infant interaction.

“It felt like we were force feeding her. That’s what it felt like. I would actually dread feeding her because I knew what was going to happen every time we fed her. As soon as it went down, she was sick, we’d wait, then she’d have a hypo.” - P3

Parents frequently described feeling compelled to encourage or complete feeds despite resistance from their infant because maintaining adequate nutritional intake was essential for glycaemic stability. This altered the emotional experience of feeding, with parents describing discomfort and distress associated with what they perceived as having to prioritise medical necessity over their child’s feeding cues.

‘We just felt like we were having to force feed her. It was awful and she had to have that amount because of her blood sugars.’ - P3.

The need to maintain blood glucose levels also influenced parents’ ability to adopt developmentally appropriate feeding practices. Several described feeling unable to follow widely recommended approaches, such as baby-led weaning, because opportunities for food exploration were outweighed by concerns that insufficient intake could result in hypoglycaemia. Parents therefore prioritised ensuring that food was consumed, even when this conflicted with their preferred approach to introducing complementary feeding.

‘I couldn’t do baby led weaning. It wasn’t something that I could do, because when you look at all of the advice and it says let them play with food. Well, I couldn’t, I had to make sure that she had to eat because we knew she would be low if she didn’t eat.’ - P9.

Across participants, feeding was conceptualised less as a responsive interaction between parent and infant and more as a therapeutic strategy for maintaining metabolic stability. Parents described how the constant need to prevent hypoglycaemia reshaped feeding decisions, often requiring them to override their infant’s cues and adapt parenting practices in ways they perceived as fundamentally different from those recommended for healthy infants.

‘He didn’t know hunger because he was never allowed to be hungry because his blood sugars were being monitored and maintained all the time. So, he just didn’t have that drive or that absolute innate hunger that a baby would have to just go.’ - P10.

### Psychosocial impact on families

Parents consistently described how the demands of feeding a child with CHI extended beyond nutritional management and became a pervasive feature of everyday family life. The continual responsibility of preventing hypoglycaemia influenced parents’ routines, decision-making and emotional wellbeing, with feeding described as a constant source of vigilance rather than a routine aspect of caring for an infant. Across participants, feeding responsibilities frequently shaped how families organised their daily lives and interacted with others.

Many parents described the practical demands of feeding as restricting family activities. Rather than leaving the house spontaneously, outings required extensive planning to ensure feeding equipment, medications and emergency supplies were available. Parents described everyday activities becoming logistically challenging because feeding was closely linked to maintaining glycaemic stability.

‘It makes it a bit harder to get out and about because now he is on blended diet as well as having his milk through a pump. So, it just means there’s no such thing as just nipping out with him. Because we always have to pre-plan which makes it harder.’ - P2.

Similarly, parents described carrying large amounts of feeding equipment whenever they left home, reflecting the constant preparedness required to safely manage their child’s condition.

‘I maybe found that I wouldn’t go somewhere as much just because of the hassle and the prep involved and leaving the house to make sure you’ve got the water to flush the line and a spare tube just in case, all the wee sticky things, pair of scissors, just in case to stick it back on and, yes, literally, even just to go to the shop, you feel like you need to have all that with you.’ - P7.

Alongside these practical challenges, parents described persistent anxiety surrounding feeding and hypoglycaemia. Rather than responding to their child’s feeding cues alone, they described continuously anticipating and monitoring the possibility of hypoglycaemia, creating an ongoing sense of responsibility that many contrasted with their experiences of caring for healthy infants.

‘I feel like it is intense, constant. Making sure he is fed. It’s like, with a normal baby you’ll listen to them, they’ll cry. But with Patient 1, he’ll probably cry because he wants a feed. But its more because is he going to have a hypo? when will he have a hypo? Constantly checking his sugars to make sure he’s not going to have a hypo, then having to manage that around feeding him.’ - P1.

For some families, feeding challenges also contributed to social isolation. Parents described avoiding public settings because of concerns about tube feeding, vomiting or attracting unwanted attention, resulting in missed opportunities to participate in parent–infant groups and establish social networks with other families.

‘It stopped me from meeting up with friends who have got children a similar age to P3. It was because of the NG tube; you would be starred at. People would be looking at her. Then she would be sick, so then she would need to be cleaned up. So, it just stopped me from going out. It just wasn’t pleasant. So, we missed out on going to baby groups and socialising meeting other families and children.’ - P3.

The emotional impact of these experiences was evident throughout the interviews. One participant became visibly tearful when reflecting on the effect of their child’s diagnosis and feeding difficulties on family life and stated, *“I don’t think I want to look at that yet.”* (P8). The interview was not pursued further on this topic to respect participant wellbeing, highlighting the emotional sensitivity of discussing the longer-term impact of feeding and disease management.

Collectively, these accounts demonstrate that the psychosocial consequences of feeding extended well beyond the act of providing nutrition. Parents described how the continual responsibility of maintaining glycaemic stability shaped daily routines, limited social participation and created persistent emotional strain, illustrating that the impact of feeding management permeated many aspects of family life.

## Discussion

This study offers a qualitative examination of the feeding experiences of families caring for children with CHI. A major strength of the study is the collection of rich, detailed insights from parents and caregivers managing the care of infants and children with CHI, explored through semi-structured interviews.

The experiences of parents and caregivers managing feeding in children with CHI highlight the multifaceted challenges inherent in the condition. Parents reported pervasive anxiety related to hypoglycaemia, feelings of guilt when strict feeding regimens or force-feeding felt necessary, and significant disruption to daily routines and social activities; however, they did not report if their anxieties were specific to any feeding type, for example breast feeding. Coordinating care across multiple healthcare providers, navigating inconsistent care plans, and managing prolonged hospital admissions added further complexity, sometimes contributing to oral aversion and feeding difficulties in children.

The emotional impact of managing CHI appeared to extend beyond the practical demands of feeding and glucose management. For some parents, reflecting on the personal consequences of their experiences remained challenging. One participant became tearful when asked about the impact of their child’s condition on their quality of life and declined to elaborate, stating, *“I don’t think I want to look at that yet.”* This suggests that, for some families, the psychological consequences of caring for a child with CHI may remain difficult to acknowledge or articulate, even when discussing their experiences in a supportive research setting.

The experiences described by parents in this study are consistent with the broader literature of paediatric feeding disorders in medically complex infant populations. Previous research has demonstrated that feeding difficulties can substantially alter the parent-child feeding relationship, increase caregiver burden, and transform feeding from a developmental and relational activity into a source of stress and anxiety (17). Similar challenges have been reported among families of children with complex medical needs, enteral feeding dependence, and chronic childhood conditions (18). However, while the experiences described by caregivers of children with CHI are similar to other conditions, they are compounded by the central role of feeding in maintaining glycaemic stability. Unlike feeding disorders in other conditions, unsuccessful feeding in CHI may be perceived as carrying an immediate risk of hypoglycaemia, increasing feelings of responsibility and pressure to ensure adequate intake. This fear clearly comes across in participant interviews. Feeding mode, including breastfeeding, may influence parental experiences; however, this was not specifically raised by parents in our cohort and consequently could not be explored in detail within this study.

Recent literature has evaluated the impact of continuous glucose monitoring on families with CHI (19–21); it is possible that CGM wear may have influenced caregivers’ experiences of feeding management. However, as this issue did not arise naturally within interviews, we have not speculated on this and would support future studies exploring the relationship between CGM use, feeding management, and caregiver wellbeing in families affected by CHI.

Parents highlighted the need for improved awareness of CHI among healthcare professionals outside specialist centres. One parent reflected, *“I think they need to get more education, because even her GP. Anytime we take her to the doctor’s or anything like that we have to explain her condition.”* This suggests that families often perceive themselves as the primary source of information when accessing non-specialist healthcare services, increasing the burden of managing an already complex condition. Unlike diabetes mellitus, which is encountered frequently across primary and secondary care, CHI is a rare disease that many clinicians may encounter infrequently, if at all. Consequently, limited familiarity with its presentation and management is understandable but may contribute to uncertainty when caring for affected children. Improving education and awareness of CHI among general practitioners, emergency clinicians and community healthcare professionals could facilitate earlier recognition, improve confidence in the management of hypoglycaemia, and reduce the need for families to repeatedly explain their child’s condition. Strengthening links between specialist CHI services and community healthcare providers may further support continuity of care and improve the experiences of families navigating multiple healthcare settings. Whether additional support from parent support groups might benefit parental feeding experiences remains unclear; this aspect was not raised by parents in interviews. Nonetheless, with greater recognition of feeding problems by CHI support groups, this would be an important area for future interviews to address.

The findings of this study highlight the importance of a coordinated multidisciplinary team approach to feeding management in infants and children with CHI. Parents described feeding challenges that extended beyond nutritional intake and encompassed oral aversion, delayed feeding development, enteral feeding dependence, complex medical regimens, and considerable psychosocial burden. Specialist CHI nurses play a central role in coordinating care, providing education and supporting families in the home environment. Dietitians are essential in optimising nutritional intake while balancing the metabolic demands of maintaining euglycaemia, and speech and language therapists contribute specialist assessment and intervention for children experiencing oral aversion and feeding difficulties. Paediatric endocrinologists oversee the medical management required to maintain glycaemic stability, while psychologists may help address the emotional burden associated with feeding difficulties and chronic disease management. Early, coordinated MDT intervention may reduce feeding-related complications, promote feeding development, and provide families with the practical and emotional support required to navigate the complex relationship between feeding and glycaemic control.

A limitation of this study is that it was conducted at a single centre, albeit one drawing from a very large section of the country. Therefore, feeding practices, clinical pathways, and family support systems may differ in alternative healthcare settings. A further limitation is the relatively small sample size and that participants were selected from a cohort of patients with confirmed genetic CHI. Families of children with milder forms of CHI are unlikely to have persistent hypoglycaemia and are less likely to experience persistent feeding problems. However, our findings would remain relevant and transferrable to other cohorts with severe forms of CHI, even those without genetic aetiology.

Our study findings accentuate that feeding in CHI extends beyond nutritional management, encompassing substantial psychological, social, and developmental dimensions. Effective care requires not only medical interventions to maintain glycaemic stability but also multidisciplinary care and integrated support addressing parents/caregiver stress, feeding strategies, and structured guidance across healthcare services. Holistic approaches that recognise both the medical and psychosocial burden of CHI are essential to optimise outcomes for children and their families.

In conclusion, this study highlights the considerable practical, emotional and social challenges associated with feeding infants and children with CHI. Feeding was frequently described as extending beyond nutrition alone and becoming closely linked to concerns regarding hypoglycaemia, medical management and parental responsibility. These findings emphasise the importance of coordinated multidisciplinary support that addresses feeding development and family wellbeing. Future research should explore interventions that reduce caregiver burden, including psychological support, peer-support initiatives and technologies such as continuous glucose monitoring, to optimise outcomes for both children with CHI and their families.

## Data Availability

The raw data supporting the conclusions of this article will be made available by the authors, without undue reservation.
